# Why do general practitioners not refer patients to behaviour-change programmes after preventive health checks? A mixed-method study

**DOI:** 10.1186/s12875-019-1028-2

**Published:** 2019-10-11

**Authors:** Nina Kamstrup-Larsen, Marie Broholm-Jørgensen, Susanne O. Dalton, Lars B. Larsen, Janus L. Thomsen, Janne S. Tolstrup

**Affiliations:** 1 National Institute of Public Health, University of Southern Denmark, Studiestræde 6, 1455 Copenhagen, Denmark; 2 Survivorship Research Unit, the Danish Cancer Society Research Center, Copenhagen, Denmark; 3 Department of Clinical Oncology and Palliative Care, Zealand University Hospital, Naestved,, Denmark; 4 Research Unit of General Practice in Odense, University of Southern Denmark, Odense, Denmark; 5 Research Unit for General Practice in Aalborg, Department of Clinical Medicine, Aalborg University Hospital, Aalborg, Denmark

**Keywords:** General practice, Behavioural support, Referral, Municipal health service, Mixed methods, Preventive, Cross-sectoral collaboration, Barriers

## Abstract

**Background:**

This study was embedded in the Check-In randomised controlled trial that investigated the effectiveness of general practice-based preventive health checks on adverse health behaviour and early detection of non-communicable diseases offered to individuals with low socioeconomic positions. Despite successful recruitment of patients, the intervention had no effect. One reason for the lack of effectiveness could be low rates of referral to behaviour-change programmes in the municipality, resulting in a low dose of the intervention delivered. The aim of this study is to examine the referral pattern of the general practitioners and potential barriers to referring eligible patients to these behaviour-change programmes.

**Methods:**

A mixed-method design was used, including patients’ questionnaires, recording sheet from the health checks and semi-structured qualitative interviews with general practitioners. All data used in the study were collected during the time of the intervention. Logistic regressions were used to estimate odds ratios for being eligible and for receiving referrals. The qualitative empirical material was analysed thematically. Emerging themes were grouped, discussed and the material was re-read. The themes were reviewed alongside the analysis of the quantitative material to refine and discuss the themes.

**Results:**

Of the 364 patients, who attended the health check, 165 (45%) were marked as eligible for a referral to behaviour-change programme by their general practitioner and of these, 90 (55%) received referrals. Daily smoking (OR = 3.22; 95% CI:2.01–5.17), high-risk alcohol consumption (OR = 2.66; 95% CI:1.38–5.12), obesity (OR = 2.89; 95% CI:1.61–5.16) and poor lung function (OR = 2.05; 95% CI:1.14–3.70) were all significantly associated with being eligible, but not with receiving referral. Four themes emerged as the main barriers to referring patients to behaviour-change programmes: 1) general practitioners’ responsibility and ownership for their patients, 2) balancing information and accepting a rejection, 3) assessment of the right time for behavioural change and 4) general practitioners’ attitudes towards behaviour-change programmes in the municipality.

**Conclusion:**

We identified important barriers among the general practitioners which influenced whether the patients received referrals to behaviour-change programmes in the municipality and thereby influenced the dose of intervention delivered in Check-In. The findings suggest that an effort is needed to assist the collaboration between general practices and the municipalities’ primary preventive services.

**Trial registration:**

Clinical Trials NCT01979107; October 25, 2013.

## Background

Preventive health checks are widely used to identify risk factors for disease and to provide early detection of metabolic risk factors in order to reduce morbidity [1–4]; however, their effects are disputed when they are offered to the general adult population [5, 6]. Targeted health checks have emerged as an alternative to the delivery of health checks to the general population [3]. In the Check-In study we examined the effect of preventive health checks on adverse health behaviour and metabolic risk factors and non-communicable diseases, targeting individuals with low levels of education in a randomised controlled design. The Check-In study was undertaken in 32 general practice clinics with 56 GPs in Copenhagen, Denmark in 2014–2016 and consisted of a preventive health check followed by a health consultation with the general practitioner (GP). Patients with abnormal results from the health checks or adverse health behaviour amenable to intervention were followed for further diagnostic work-up and medical treatment and/or referred a to behaviour-change programme at the municipal health services, offered free of charge. Further, patients with metabolic risk conditions such as hypertension, high cholesterol levels or low lung function could be referred to targeted behaviour-change programmes in the municipality. Even though the recruitment strategy in Check-In seemed successful in reaching the target group [7], the intervention had no effect on adverse health behaviour, levels of metabolic risk factors or incidence of non-communicable diseases [8].

Process evaluation, including evaluation of the ‘dose delivered’ component, can help explain the lack of effect of public health interventions and improve understanding [9]. The Check-In intervention was tested in a real-life setting and the intervention was delivered by GPs in demanding working environments. In Check-In, the health consultation after the preventive health checks was a crucial point in the implementation of the intervention, and important for the dose of the intervention delivered in Check-In. At the health consultation the GPs first decided whether the patient was eligible for referral and then whether to actually refer or not, in consultation with the patient. One might hypothesise that the dose of intervention delivered was too low to produce an average effect size in the intervention group that was significantly different from that of the control group. In the present study we therefore examined the ‘dose delivered’ of the Check-In intervention to those individuals allocated to the intervention group. We did this by analysing the referral pattern of the GPs and potential barriers to referring otherwise eligible patients to behaviour-change programmes after preventive health checks.

### Theoretical perspective

The decision-making process is based on an uneven distribution of knowledge, as the GPs hold the power of the superior knowledge in the clinical encounter [10]. Norwegian Philosopher Harald Grimen argues that this power cannot be utilised unless the patient trusts the GP and the professional risk perspective [10]. This means that the assessment of whether the patient receives a referral to the municipal health services is not exclusively based on the GP’s professional knowledge. Examining the GPs’ assessment, we draw on Tim Rapley’s dynamic outline of decision-making as an ongoing event that goes beyond the individual encounter and often evolves over multiple encounters [11]. In this view, decision-making is a process that is distributed across time, people and sources of knowledge. This approach aligns well with general practice, where the patient list system allows for a continuous relationship between GPs and patients, as patients often see the same GP for many years [12]. We apply this theoretical framework of the clinical encounter to explore and understand GPs’ assessment of whether to refer patients to behaviour-change programmes in the municipality.

### The check-in study – design and setting

To identify the study population in Check-In (individuals without formal education beyond lower secondary school), questionnaires were sent to patients between 45 and 64 years of age from the participating GPs’ patients list. Individuals who reported no formal education beyond lower secondary school and indicated in the questionnaire that they were willing to be contacted for further research were enrolled in Check-In. In total, 549 individuals were allocated to the intervention group and 555 were allocated to the control group. The intervention group received a personal postal invitation to a prescheduled preventive health check from their GP. The health checks took place at the general practice clinics where the patients were registered and included measurements such as weight and height, blood pressure, a blood sample for measuring serum cholesterol and spirometry. Approximately two weeks after the health check, the patient had a health consultation with the GP. Here the GPs reviewed the results from the health checks and the patient-reported questionnaires. Additionally, the need for referrals to behaviour-change programmes and/or further diagnostic work-up and medical treatment was discussed. Individuals allocated to the control group received the usual care during the intervention period. General practice was used as setting in Check-In because of the GPs’ function as gatekeepers for the rest of the healthcare system [13]. This gatekeeper function is characteristic of general practice in Denmark, as well as in many other countries. This specific role of the GPs ensures that they are often the first point of contact in the health care sector [13]. Further, it means that GPs are in contact with the majority of the population and often over prolonged periods of time, which gives general practices a natural potential for incorporating prevention and health promotion [14]. GPs are responsible for ensuring easy access to healthcare for everyone and they may refer patients to behaviour-change programmes in the municipal setting [15]. In this way, the Check-In study took advantage of the pre-existing behaviour-change programmes in the municipal setting using the existing referral process. Because the referral process is part of usual care, the GPs received no training in this process. However, at the beginning of Check-In the project group presented the different offers available in the municipality to the GPs and outlined the referral process.

## Material and methods

This paper reports on an emergent mixed method study with a convergent design [16]. Hence, the specific study aim and the hypothesis were not planned or given prior to the data collection, but emerged as a result of the lack of effect on adverse health behaviour, metabolic risk factors and non-communicable diseases. However, all data used in the study were collected at the time of the intervention. The availability of both quantitative and qualitative data provided an opportunity to get a deeper understanding [17] of the referral behaviours of the GPs and the barriers to referral than would have been possible with only the quantitative or qualitative data. The mixed method study comprises three main data sources: patient questionnaires, GP recording sheets and semi-structured interviews. Patient questionnaires and recording sheets were used to examine GPs’ referral patterns, whereas the semi-structured interviews were used to elicit barriers to referral to behaviour-change programmes.

### Patient questionnaires

Of the 549 individuals allocated to the Check-In intervention group, 364 (66%) attended the health check and were included in the present study. Information about adverse health behaviour and BMI was obtained from the baseline questionnaires. Smoking status was dichotomised into smokers versus non-smokers. Alcohol consumption was reported for a normal week and high-risk alcohol consumption was defined according to national recommendations as more than 14/21 units of alcohol per week for women and men, respectively [18]. BMI was generated from the self-reported height and weight and categorised using BMI ≥30 as a cut point for obesity [19]. Physical activity was dichotomised into ‘low or physically inactive’, defined as less than 150 min of adequate or high level physical activity throughout the week, less than 75 min of vigorous-intensity physical activity throughout the week or an equivalent combination of moderate- and vigorous-intensity activity defined as ‘adequate or high’, as defined by the WHO [20]. Moreover, self-efficacy, measured by the general self-efficacy scale [21, 22], and perceived stress, measured by Cohen’s 10-item Perceived Stress Scale [23], were analysed as continuous variables.

### National administrative registers

Using the unique personal identification numbers assigned to all residents in Denmark, we linked the questionnaire survey to the Danish Civil Registration System to obtain information about country of origin and marital status [24]. Information on employment status was obtained from the Employment Classification Module obtained from Statistics Denmark and categorised as unemployed or employed [25].

### Project-specific health-check recording sheet

In the recording sheets the GPs marked if the patient was already being monitored in the clinic and if it was relevant to refer the patient to behaviour-change programmes. If the patient received a referral, the GP stated which programme/programmes the patient was referred to (smoking cessation, conversations about excessive alcohol consumption, guidance on diet, guidance on exercise, disease specific health promotion/rehabilitation). If the patient was not referred to a behaviour-change programmes, the reasons were noted. In addition, the GPs noted the results from the health checks (blood pressure, total cholesterol, and spirometry). Blood pressure level was characterised as hypertension or normal, with hypertension being defined as a blood pressure level of > 140/90 mmHg. Total cholesterol was dichotomised into high cholesterol, defined as > 5 mmol/l, and normal cholesterol as below this level. From the spirometry FEV1 and FVC were used to calculate the ratio and the variable was dichotomised as FEV1/FVC < 70% or above.

### Semi-structured interviews

All GPs who participated in the Check-In RCT were invited by email to participate in the interviews. The email provided information about the aim of the interview, expected dissemination, length, fee, use of a dictaphone and anonymity. Having already recruited the GPs to participate in Check-In made the recruitment to the interviews easier as GPs were eager to discuss their experiences with Check-In. None of the GPs actively declined to participate. Due to time constraints and data saturation, a sample of 17 GPs was included. They participated in the semi-structured qualitative interviews (Table 1).

**Table 1:** Characteristics of interviewed general practitioners

GP	Sex (M=male/F=female)	Age (years)	Character of clinic	Number of GPs in the clinic
A	F	60–64	Solo surgery	1
B	F	50–54	In partnership	3
C	M	60–64	Solo surgery with shared facilities	3
D	M	55–59	Solo surgery	1
E	M	40–44	In partnership	2
F	M	60–64	Solo surgery with shared facilities	2
G	F	50–54	In partnership	2
H	F	40–44	In partnership	4
I	F	55–59	Solo surgery	1
J	F	40–44	In partnership	3
K	F	40–44	In partnership	3
L	F	40–44	In partnership	2
M	F	< 40	Solo surgery with shared facilities	2
N	M	40–44	In partnership	2
O	M	55–59	Solo surgery	1
P	M	45–49	Solo surgery with shared facilities	2
Q	M	50–54	Solo surgery	1

The sample strategy was maximum variation regarding sex, age and type of practice. Overall, the sample of GPs matched the population of GPs in the capital region of Denmark in terms of sex, age and practice type at the time of the Check-In study. With the aim of exploring the GPs’ reasons for offering referrals to behaviour-change programmes, we applied an explorative approach [17] that involved examining the GPs’ experiences with assessing patients’ eligibility for referral and providing the referral. MB-J conducted the interviews between 2013 and 2015. The interviews were structured around the themes of participation in the Check-In study, perceptions of risk and experiences with preventive health checks in clinical encounters. The interviews lasted approximately one hour and were conducted in the GPs’ consultation room. The interviews with the GPs were digitally recorded and transcribed verbatim by MB-J.

#### Analysis

**Fig. 1:**
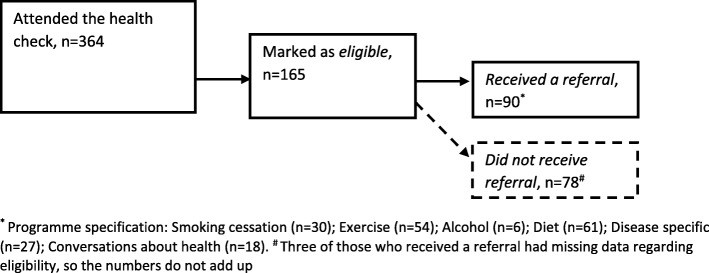
Relationship between attendees, eligible patients and patients receiving referral

Receiving a referral was a two-step procedure. First, the GPs had to find the patient eligible and second, the patient and the GPs had to agree on the referral. Two groups of patients were identified from the recording sheets: 1) patients eligible for referral and 2) patients who received a referral*.* Patients were defined as eligible if the GPs in the recording sheet answered “yes” to the question: “Is it relevant to refer the patient to behaviour-change programmes in the municipality?”. The eligible patients were further divided into two groups: those who received a referral and those who did not receive a referral (Fig. 1).

Baseline characteristics were reported for 1) all patients who attended the health check, 2) the eligible patients, 3) the patients who received a referral and 4) eligible patients who did not receive a referral. Logistic regressions were used to estimate odds ratios (ORs) for being eligible and for receiving a referral, respectively. Sample size calculations were not performed for these specific analyses; thus, the results had an exploratory character and analyses were unadjusted due to the small sample size. Results for self-efficacy and perceived stress were calculated using continuous scores but were also tested as dichotomised variables using the median split. ORs were estimated for each specific adverse health behaviour and BMI as well as for a categorical variable. The categorical variable was generated by counting (yes = 1, no = 0) daily smoking, high-risk alcohol consumption, obesity and low physical activity and divided into 0, 1 or ≥ 2 adverse health behaviours or obesity. The same was done for metabolic risk conditions.

The analysis of the qualitative empirical material followed principles for qualitative analysis [17]. All transcriptions were initially read by NK-L and MB-J with a broad focus on experiences and perspectives on referring patients to the municipal health services. Based on the initial reading, themes were identified, such as *assessing the right time for behavioural change* and *expectations about the effectiveness of the behaviour-change programmes in the municipality,* after which the empirical material was revisited. In the analytical process that followed, the themes were reviewed alongside the analysis of the baseline questionnaires and recording sheets in order to refine and discuss the themes that emerged from the qualitative material. The preliminary results were discussed in the context of existing literature on decision-making and assessment of risk in in the clinical encounter. Quotes were selected and used in the results section to illustrate key themes as raised by the GPs.

## Results

### GPs’ referral behaviour

Of the 364 patients who attended the health check, 165 (45%) were marked as eligible for a referral to behaviour-change programmes (Fig. 1). However, not all smokers, not everybody who exceeded the high-risk alcohol limit and not all obese individuals were marked as eligible. Still, daily smoking (OR = 3.22; 95% CI:2.01–5.17), high-risk alcohol consumption (OR = 2.66; 95% CI:1.38–5.12), obesity (OR = 2.89; 95% CI:1.61–5.16) and having a FEV1/FVC < 70% (OR = 2.05; 95% CI:1.14–3.70) were statistically significant associated with being eligible. ORs for being eligible increased with higher number of adverse health behaviours. Moreover, we found that employed patients were less likely to be eligible when compared to unemployed (OR = 0.49; 95% CI:0.31–0.77) (Table 2). Of the 165 eligible patients, 90 received a referral (55%) (Fig. 1). Women were significantly more referred when compared to men (OR = 2.78; 95% CI:1.48–5.22), while individuals of Western origins were less likely to be referred when compared to individuals of non-Western origins (OR = 0.33; 95% CI:0.13–0.80). Of the 135 daily smokers, 30 (22%) received a referral to smoking-cessation programmes, and of the 50 high-risk drinkers, 6 (12%) received a referral to alcohol treatment in the municipality (data not shown). We found no association between daily smoking, high-risk alcohol consumption, obesity, low FEV1/FVC or combinations of these factors and actually receiving a referral to the behaviour-change programmes. Neither self-efficacy nor perceived stress were associated with being eligible or being referred (Table 2).

**Table 2:** Characteristics for attendees, eligible patients and those who received a referral

	Attendees of the health check; *n* = 364	Eligible for referral; *n* = 165	OR for eligible	Eligible for referral		
				Received a referral; *n* = 90	Did not receive a referral; *n* = 78	OR for received a referral
Demographic and socioeconomic	N (%)	N (%)	OR (95% CI)	N (%)	N (%)	OR (95% CI)
Sex						
Male	181 (50)	89 (54)	1	36 (40)	51 (65)	1
Female	182 (50)	76 (46)	0.81 (0.53–1.25)	54 (60)	27 (35)	2.78 (1.48–5.22)
Cohabitation status						
Living without partner	155 (43)	74 (45)	1	43 (48)	33 (42)	1
Living with partner	208 (57)	91 (55)	0.80 (0.52–1.24)	47 (51)	45 (58)	0.78 (0.42–1.45)
Origin country						
Non-western origin	68 (19)	36 (22)	1	23 (26)	8 (10)	1
Western origin	296 (81)	129 (78)	0.79 (0.46–1.36)	67 (74)	70 (90)	0.33 (0.13–0.80)
Employed status						
Unemployed	128 (35)	69 (42)	1	41 (46)	31 (40)	1
Employment	235 (65)	96 (58)	0.49 (0.31–0.77)	49 (54)	47 (60)	0.79 (0.43–1.46)
Self-reported adverse health behaviour and health status						
Daily smoking						
No	222 (61)	80 (49)	1	50 (56)	34 (44)	1
Yes	135 (37)	80 (49)	3.22 (2.01–5.17)	37 (41)	44 (56)	0.57 (0.31–1.06)
High risk alcohol consumption (14/21 units)						
No	288 (79)	120 (73)	1	73 (81)	53 (68)	1
Yes	50 (14)	34 (21)	2.66 (1.38–5.12)	13 (14)	19 (24)	0.50 (0.23–1.09)
Sedentary or low physical activity						
No	195 (53)	82 (50)	1	42 (47)	47 (60)	1
Yes	167 (46)	83 (50)	1.36 (0.89–2.10)	48 (53)	31 (40)	1.73 (0.94–3.20)
Obesity (BMI ≥ 30)						
No	278 (76)	113 (68)	1	60 (67)	57 (73)	1
Yes	73 (20)	45 (27)	2.89 (1.61–5.16)	27 (30)	17 (22)	1.51 (0.74–3.06)
Number of adverse health behaviours (including obesity)						
0	89 (24)	19 (12)	1	14 (16)	9 (12)	1
1	113 (31)	51 (31)	3.08 (1.62–5.85)	24 (27)	30 (38)	0.51 (0.19–1.39)
≥ 2	118 (32)	74 (45)	7.55 (3.90–14.61)	43 (48)	29 (37)	0.95 (0.36–2.49)
Self-efficacy [median]	30 [25;33]	29 [23;33]	0.97 (0.94–1.00)	27 [23;32]	30 [24.5;34]	0.98 (0.93–1.02)
Perceived stress [median]	15 [11;20]	16 [11;20]	1.01 (0.98–1.05)	17 [12;21]	15 [10;20]	1.04 (0.99–1.09)
Results from the health checks						
Hypertension (> 140/90)						
No	251 (69)	105 (64)	1	57 (63)	49 (63)	1
Yes	113 (31)	60 (36)	1.70 (1.06–2.72)	33 (37)	29 (37)	0.98 (0.52–1.83)
Total cholesterol > 5 mmol/l						
No	115 (32)	55 (33)	1	28 (31)	27 (35)	1
Yes	206 (56)	96 (58)	0.90 (0.56–1.44)	55 (61)	42 (54)	1.26 (0.65–2.45)
FEV1/FVC < 70%						
No	256 (70)	112 (68)	1	62 (69)	48 (62)	1
Yes	63 (17)	38 (23)	2.05 (1.14–3.70)	19 (21)	21 (27)	0.70 (0.34–1.45)
Number of metabolic risk conditions						
0	52 (14)	19 (12)	1	9 (10)	10 (13)	1
1	154 (42)	76 (46)	1.85 (0.96–3.58)	45 (50)	28 (36)	1.79 (0.65–4.94)
≥ 2	79 (22)	42 (25)	2.08 (1.00–4.31)	21 (23)	23 (29)	1.01 (0.35–2.98)

N(%) if nothing else is stated

Crude odds ratios (95% confidence intervals) for being *Eligible* and for *Receiving a referral*

### GPs’ referral barriers to behaviour-change programmes

In the following, we report on barriers to referring eligible patients to behaviour-change programmes. The analysis identified four themes: 1) *GPs’ responsibility for and ownership of their patients*, 2) *balancing information and accepting a rejection*, 3) *assessment of the right time for behavioural change* and 4) *GPs’ attitudes towards behaviour-change programmes at the municipality.*

### GPs’ responsibility for and ownership of their patients

The GPs felt responsible for their patients, reporting that they wished to protect the patients from just another failure by considering their everyday life. The GPs demonstrated a sense of responsibility and ownership of their patients’ health and wellbeing:

*MB-J: They’re your patients, I guess? (laughs)*

*GP B: Yes, they’re ours. So, we have to take care of them. And we also have a responsibility to make sure that they receive the treatment they need.*

This also involved protecting the vulnerable patients from experiencing failure in their attempts to change their adverse health behaviour:

*They [the patients] might not have the resources or surplus of mental resources to do anything about it. So, it just turns out to be another failure if they have to sit and listen to “If you don’t stop smoking then you risk that and that”. And of course, some help will also be offered, but I don’t really think they need to be confronted with their problems. It’s like if you’re a bad [bill] payer and you hide your bills in the drawer so they’re out of sight out of mind.* (GP E)

This illustrates that in the assessment of whether to refer to behaviour-change programmes, the GPs include knowledge about the patient and reflections about the patients’ personal resources and interests in talking about e.g. smoking cessation.

### Balancing information and accepting a rejection

The GPs reported that offering patients referrals to behaviour-change programmes involved finding a balance between informing patients about the opportunity and at the same time expressing understanding and acceptance of a possible rejection of the offer. Failure to keep this balance could lead the GPs to appear angry or offended, which according to the GPs could make patients worry that they would not receive the offer again if and when they felt ready to change their adverse health behaviour.

*The GPs can easily be perceived as annoyed and arrogant, or as if they’re rebuffing the patients without really meaning to. If I say, “I have a good smoking cessation offer for you in the municipality”, and the patient then says “no”, then the GP might accidentally say “hmm” or withdraw with their body language and in that way show a dismissive attitude. And then the patient won’t have the courage to bring it up again at a different time. Well it can be completely unintentional on the GP’s part, but it might make the patient say [to himself], “Now the GP got offended, and now I’ve said no, I can’t bring it up again. Well, now that door is likely closed”. Then I think it it’s important to say, “The offer stands if you change your mind. It’s here. You can just let me know. I won’t be offended”. There are patients who ask me, “Will you be offended if I say no?”. “No, no, no, after all this is about your health. You can decide for yourself.” And then [they] know that the offer will always be there. We can always talk about it.* (GP L)

According to this GP, the offer of a referral should be presented in a way that ensure the patients can turn to the GP when it is relevant. The GPs emphasised the importance of balancing the information provided to patients they assessed as lacking motivation for behavioural change*.* The awareness of the balance in the information could result in non-referral of some eligible patients. Additionally, several of the GPs expressed concerns about putting too much pressure on the patients:

*GP F: There was also somebody who said, was aware that there was something here that was not so good and the said, “But I can’t be bothered now. It’s not now I need to do it”*

*[ … ]*

*MB-J: What do you say then?*

*GP F: “That’s completely okay. I can understand if you don’t have time for it, but at some point you might get time for it and then you can just come back”. Don’t put pressure on [the patient], don’t put pressure on [the patient].*

*MB-J: Don’t pressure them, but show*

*GP F: that you’re open.*

Respecting and recognising patients’ choices by not pressuring them to accept the referral in the present encounter was thus perceived as a strategy to inform patients about the offer of help to change adverse health behaviour when the right time occurred.

### Assessment of the right time for behavioural change

**Fig. 2:**
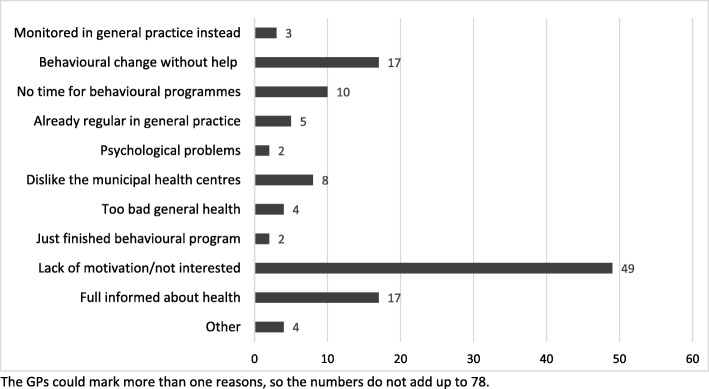
Reasons not to refer *eligible* patients to behaviour-change programmes at the municipal

Assessing whether it was the right time for behavioural change for the patients, acted as a barrier and most likely affected who the GPs offered a referral to. The patients’ age and employment status made the GPs anticipate that patients would not be interested in or motivated for behaviour-change programmes. Among the eligible patients who did not receive a referral (*n* = 78), the recording sheets showed that the most common reasons given by the GPs were patients’ perceived lack of motivation (*n* = 42), that the patient already felt informed about their own health (*n* = 17), that the patient wanted to manage their behavioural change themselves (n = 17) and that the patients did not have time for behaviour-change programmes (*n* = 10) (Fig. 2). In line with this, the qualitative material showed that the GPs’ assessment of the patients’ motivation for behavioural change influenced whether the GPs offered a referral. Identifying the patients’ motivation was articulated as an assessment of whether it was the right time in the patients’ life. According to the GPs, deciding whether it was ‘the right time’ was based on different aspects such as employment status, age, adverse health behaviour and potential diagnosis or risk of non-communicable diseases. However, the assessment was not always correct. One GP described being surprised by a patient who accepted a referral to a behaviour-change programme and completed the specific programme:

*GP F: I had a young guy, I don’t remember his name, who I actually thought was fit and healthy and he got pretty excited about it [the programme]. I referred him, and he went to the municipal health centre here. Yes, he jumped right in and was really pleased about it.*

*[ … ]*

*GP F: I thought “No, he doesn’t have time for that, a young guy like that”, because he was relatively young. “He won’t have the time for that, he won’t do it”. That surprised me, I have to say. And he had achieved something from it. Something measurable so to speak.*

*MB-J: And you reviewed these things with him afterwards?*

*GP F: Yes, yes, so that can you say, “Why now?”. It [the offer] might have come at the right time in his life. But well what the hell. It was fine, I think.*

The GPs also stated that some patients were well under-way with changing health behaviour or had already made significant changes and hence would not be referred despite being eligible.

### GPs’ attitudes towards behaviour-change programmes at the municipality

The decision-making regarding referral to behaviour-change programmes was not exclusively based on the professional knowledge, as a scepticism towards the effectiveness of these programmes was common among the GPs and acted as a barrier for the GPs to referring the patients. Overall, this scepticism related to an assumption that the preventive message is widespread in the population and that adding additional information would not make these patients change their health behaviour:

*Well, there were lots of things to tackle, it was not that at all, but we knew that beforehand and we know that about this patient group. Instead I think that one should discuss whether it would be of any use, because who the hell doesn’t know that it’s dangerous to smoke and drink too much? Everyone knows that. Will it be of any use to refer them to the behaviour-change programmes at the municipality when they weigh too much? I think it costs a lot for each kilo people lose by sending them there. So, I think maybe one should consider whether it's even useful to find those things. I'm not a big fan of too much outreach work. I honestly believe that it doesn't really help anything.* (GP E)

According to the GPs, patients, including patients with low levels of education, were well aware of the consequences of adverse health behaviour, and therefore the GPs questioned the effect of referring the patients to behaviour-change programmes which reflected their perception of the programmes on offer as purely informative. The GPs’ reasons for not providing these otherwise eligible patients with a referral was that the programmes “*Wouldn’t teach the patients anything they didn’t already know*” (GP L).

However, despite the fact that referring patients to the behaviour-change programmes in the municipality was not specific to the Check-In study, just the possibility of referrals was emphasised as an advantage in Check-In:

*So that was in a way very positive, because this is what is the good about this kind of study. We have something to offer. So, we have some prevention offers that we can refer them to.* (GP D)

## Discussion

### Main findings

We found that only around half of those marked as eligible for behaviour-change programme were eventually referred. Hence, the dose of intervention delivered in Check-In was lower than anticipated and may partly explain the lack of effect from the intervention on smoking prevalence and other adverse health behaviours. We found that adverse health behaviour or the presence of metabolic risk conditions such as hypertension, hypercholesterolemia or low lung function were not the only indicators in the assessment of the eligibility for a referral to behaviour-change programmes and further, for receiving a referral. Perceived lack of motivation was the most prevalent reason given by the GPs for not referring a patient to a behaviour-change programme, whereas four attributes of the GPs might further explain the pattern of referrals we saw in the Check-In study: *responsibility for and ownership of their patients*, *balancing of information and acceptance of a rejection, assessment of the right time for behavioural change,* and *attitudes towards behaviour-change programmes.*

### Interpretation of findings and relation to other studies

GPs participating in the Check-In study acknowledged the importance of offering the target group of Check-In an extra effort in the preventive work. However, only 22% of the daily smokers and 6% of the high-risk drinkers were referred to behaviour-change programmes. Nevertheless, these low rates of referrals from GPs to behaviour-change programmes have been reported elsewhere. In the UK, the referral rate of eligible individuals from general practices were 4% for adult weight management [26], 7% for smokers and 34% for low-risk alcohol consumption [27]. These low referral rates highlight the fact that decision-making in referring patients to behaviour-change programmes goes beyond adverse health behaviour – and beyond the individual encounter, as suggested by Tim Rapley’s dynamic definition of the decision-making process [11]. Daily smoking, high-risk alcohol consumption, obesity and poor lung function were not associated with receiving a referral and one might wonder whether the GPs felt adequately trained to discuss these sensitive topics with the patients. However, our findings suggest that the continuous relationship and the general knowledge about the patients was of great importance to the GPs when deciding whether to offering a referral or not – and sometimes more important than the specific adverse health behaviour. This is in line with previous studies finding [28] that GPs apply the background knowledge of the patient, the patient’s current clinical presentation and the GPs’ personal opinions when deciding whether or not to refer a patient with chest pain [29]. Likewise, a recent study showed that GPs’ considerations for or against providing emergency department referrals went beyond considerations of medical surgery [30].

We found that the *GPs’ feelings of responsibility towards their patients* acted as a barrier and raised a paradox in relation to the decision-making in the clinical encounter. The GPs reported protecting their patients from experiencing failure, and if the patients were assessed as lacking personal resources for behaviour change, the GPs did not refer the patients. Besides being attentive to the patients’ resources, this could additionally indicate a concern for not harming the relationship with the patients. Several studies report GPs being concerned about harming the GP-patient relationship when giving preventive health advice [28, 31]. In prevention, GPs balance authority and respect for patients’ autonomy by compromising or sidestepping certain health issues to avoid harming the GP-patient relationship, which can have consequences for prevention in the clinical encounter [31, 32]. This may indicate that the nature of the relationship between the GP and patient influence whether a referral is offered [10].

The findings suggest that GPs struggled with *balancing information and accepting a rejection* when referrals were offered. In general, GPs’ focus on treatment is found to affect whether prevention is introduced in the clinical encounter [33]. In order to fit prevention to the clinical encounter it has been suggested to connect this with treatment – for instance, both GPs and patients find that advice on smoking cessation in general practice fits naturally with smoking-related illnesses or symptoms such as coughs [34, 35]. Studies on smoking cessation point out that people who smoke tend to accept and understand that GPs initiate discussions about smoking [36]. However, preventive advice may be tolerated more if the advice is provided by a GP with whom the patients have established a trusting relationship [37, 38]. Health checks have been suggested as suitable situations to bring up sensitive topics such as smoking and alcohol [28]. However, even in a setting like the one in Check-In with the preventive health checks before the health consultations, which potentially could point to health issues amenable to intervention, the GPs struggled with finding the best way to offer a referral. We found that the GPs’ concerns about pressuring their patients into behavioural change influenced whether the patients received referrals or not. GPs emphasised the importance of ensuring that the patients knew that they could return to the general practice when necessary, ensuring an ongoing relationship between the GPs and their patients [31]. In this way, the decision-making regarding referrals to behaviour-change programmes went beyond the individual encounter and was a dynamic process, in line with Tim Rapley’s description [11].

*Assessment of the right time for behaviour change* played an essential role in deciding whether the patients were referred or not. Patients’ lack of motivation was indicated as the main reason for not providing referrals to behaviour-change programmes. In line with this, previous research shows that GPs’ assessment of patients not being motivated to attend a health-promotion programme resulted in 37% of the eligible patients not being referred [39]. Allowing patients to turn to the GPs when the right time arose indicated that providing referrals to patients in general practice is a processual practice. Most likely this assessment of the right time affected the number of patients marked as eligible and the number of patients receiving a referral and thereby affected the dose of intervention delivered in Check-In.

Our findings show that *GPs’ attitudes towards behaviour-change programmes in the municipality,* influenced whether they referred their patients or not. Some GPs indicated that they were sceptical about the effect of the behaviour-change programmes, which may have affected whether they offered their patients referrals. However, we wonder if this scepticism is grounded in insufficient information about the offers available in the municipality. We base this assumption on our experience with presenting the participating GPs with information about the offers in the municipality in the start-up phase of Check-In. We found that most of the GPs showed little knowledge of these offers and appreciated the laminated guide that presented the offers, which was printed and handed out in connection with the Check-In study. Hence, we wonder whether the GPs’ assessment of whether the behaviour-change programmes could be relevant to the patients would have changed had the GPs had a greater knowledge about the specific offers in the municipality. In addition, the GPs argued that the preventive message is widespread in the population, including in the target group of Check-In. In some cases, the GPs believed that patients were well informed about e.g. the side effects of smoking and therefore could not see the relevance of a referral. Attitudes towards smoking have changed in the last decades and public information on smoking cessation and the harm of smoking have increased during this period. This may be an argument for the fact that in relation to smoking cessation the so-called ‘low-hanging fruit’ no longer exists and that most people are well aware of the risks associated with smoking. However, individual and general advice have very different implications [34], indicating that public information on the harm caused by smoking is not equal to smoking cessation advice from GPs in the context of health checks. Also, it is important to acknowledge that a potential smoking cessation programme includes other aspects than just information, meaning that despite the assessment of informed patients a referral to behaviour-change programmes could still be relevant.

This paper outlines barriers to the GPs referring patients to behaviour-change programmes in the municipality, which influenced the implementation of Check-In. The identified barriers are central in the GPs’ role in prevention of non-communicable diseases and are therefore noteworthy both in future studies in the setting of general practices and in the GPs’ role in prevention in general. It is, however, important to acknowledge that “no referral” does not necessary mean “no further action”. Instead of referring to behaviour-change programmes at the municipality, GPs may prefer to offer help with behaviour change themselves based on their ongoing relationship with the patient. If the patient was not deemed to be ready for behavioural change, the GP saw his or her responsibility as potentially preparing the patients to change their attitude towards behaviour change in the longer run.

### Limitations

A potential limitation might be that the interviewed GPs constituted a selected group of GPs. The 17 GPs interviewed in the present study all participated in the Check-In study. However, the recruitment of the GPs to the Check-In study was challenging, and a large proportion of the invited GPs did not want to participate in the trial – mainly because of lack of time and resources or due to a general opposition to preventive work in the clinical encounter. Nevertheless, if the included GPs experienced barriers to referring patients to behaviour-change programmes after preventive health checks, nothing indicates that this would not also be the case for the GPs who declined to participate in Check-In. GPs less interested in prevention than the participating GPs most likely would have referred fewer patients to the behaviour-change programmes. Another limitation is the small sample size and the lack of sample calculation, which meant that the results had an exploratory character. Further, it can be argued that the dichotomous decision to refer or not to refer may simplify the measurement of the ‘dose delivered’ and that a more comprehensive measure of what happens in the clinical encounter would have been desirable. However, such data were not available in the present study.

### Implications for practice and future research

For more than 10 years the Danish municipalities have been charged with the responsibility for local disease prevention and health promotion [15]. The findings in the present study indicate that the collaboration between general practices and municipalities remains challenged, as seen in previous research [40]. Even in a trial such as Check-In, in which the GPs were updated about the specific programmes offered in the municipal setting, many eligible patients were not referred. As the healthcare system is put under increasing strain, the collaboration between general practices and the municipal health services becomes increasingly important. Especially, taking into account that the time allocated to each clinical encounter is limited, only leaving time for the most necessary interventions, which is often treatment – both for patients and the GPs – with lower priority given to prevention [41]. Further research is needed to gain a better understanding of the barriers to prevention in general practice and the collaboration between general practices and the municipal health services. Observations of the clinical encounter and interviews with the patients could add information on the decision-making regarding referrals to behaviour-change programmes in the municipality. Future research could include the patients’ perspectives and investigate whether lack of motivation is as common as the GPs believe. Although both observations and interviews with the participating patients were conducted during the Check-In study, the focus of these was not on the decision-making regarding referral and therefore they were not included in the present study. Another avenue for further research could be a longitudinal study that investigates whether those without a referral received direct support from the GP or referral at a later encounter.

## Conclusion

We identified important barriers among the GPs which influenced whether the patients received referral to behaviour-change programmes in the municipality and thereby influenced the dose of intervention delivered in Check-In. We found that only half of all eligible patients received a referral to behaviour-change programmes and that adverse health behaviour or the presence of metabolic risk conditions were not the only indicators in the assessment of the eligibility, and for receiving a referral. The findings indicate that decision-making in referring patients to behaviour-change programmes goes beyond adverse health behaviour – and beyond the individual encounter. Future studies in the setting of primary care can take advantage of these findings when preparing interventions that are implemented by GPs. Further, the findings suggest that an effort is needed to encourage and assist the collaboration between general practices and the municipal health services.

## Data Availability

The quantitative data that support the findings of this study are available from Statistic Denmark, but restrictions apply to the availability of these data, which were used under license for the current study, and are not readily publicly available. Data are, however, available from the authors upon reasonable request and with permission of Statistics Denmark.

## Abbreviations

95% CI: 95% confidence interval
FEV1: forced expiratory volume in one second
FVC: forced vital capacity
GP: general practitioner
OR: odds ratio
